# Cerebrovascular disease in ageing and Alzheimer’s disease

**DOI:** 10.1007/s00401-015-1522-0

**Published:** 2015-12-28

**Authors:** Seth Love, J. Scott Miners

**Affiliations:** Institute of Clinical Neurosciences, School of Clinical Sciences, Learning and Research Level 2, Southmead Hospital, University of Bristol, Bristol, BS10 5NB UK

**Keywords:** Cerebrovascular disease, Alzheimer’s disease, Ageing, Hypoperfusion, Myelin proteins, Endothelin-1

## Abstract

Cerebrovascular disease (CVD) and Alzheimer’s disease (AD) have more in common than their association with ageing. They share risk factors and overlap neuropathologically. Most patients with AD have Aβ amyloid angiopathy and degenerative changes affecting capillaries, and many have ischaemic parenchymal abnormalities. Structural vascular disease contributes to the ischaemic abnormalities in some patients with AD. However, the stereotyped progression of hypoperfusion in this disease, affecting first the precuneus and cingulate gyrus, then the frontal and temporal cortex and lastly the occipital cortex, suggests that other factors are more important, particularly in early disease. Whilst demand for oxygen and glucose falls in late disease, functional MRI, near infrared spectroscopy to measure the saturation of haemoglobin by oxygen, and biochemical analysis of myelin proteins with differential susceptibility to reduced oxygenation have all shown that the reduction in blood flow in AD is primarily a problem of inadequate blood supply, not reduced metabolic demand. Increasing evidence points to non-structural vascular dysfunction rather than structural abnormalities of vessel walls as the main cause of cerebral hypoperfusion in AD. Several mediators are probably responsible. One that is emerging as a major contributor is the vasoconstrictor endothelin-1 (EDN1). Whilst there is clearly an additive component to the clinical and pathological effects of hypoperfusion and AD, experimental and clinical observations suggest that the disease processes also interact mechanistically at a cellular level in a manner that exacerbates both. The elucidation of some of the mechanisms responsible for hypoperfusion in AD and for the interactions between CVD and AD has led to the identification of several novel therapeutic approaches that have the potential to ameliorate ischaemic damage and slow the progression of neurodegenerative disease.

## Introduction

The relationship between age-associated cerebrovascular disease (CVD) and Alzheimer’s disease (AD) is complex. They share multiple risk factors and overlap more often than not, to an extent that increases with age. Whilst there is clearly an additive component to the clinical and pathological effects of CVD and AD, increasing evidence suggests that the disease processes also interact mechanistically at a cellular level, probably in a manner that exacerbates both. However, partly because it has proven difficult to model either of these diseases accurately individually, let alone in combination, it has been difficult to determine the extent of that interaction or to establish which of the many potential mechanisms are biologically relevant.

This review covers some of the evidence of pathophysiological interaction between CVD and AD and considers possible mechanisms and therapeutic implications.

## Clinical and pathological associations between CVD and AD

CVD and AD share many risk factors, including *APOE* ε4 [102, 141]; diabetes mellitus [139]; atrial fibrillation [38, 123]; ‘non-Mediterranean’ diet [87]; hyperhomocysteinaemia [92]; midlife hypertension, obesity and hypercholesterolaemia [30, 104, 160]; and, of course, age. The presence of vascular risk factors was reported to predict the development of AD or the conversion from mild cognitive impairment (MCI) to AD [35, 61, 90, 99]. Not all studies have found an association between vascular risk factors and AD [31, 134], and there are several potential confounders that should be considered in interpreting studies of shared risk factors or overlapping pathology, particularly as CVD and AD also overlap clinically. Concurrent ischaemic cerebrovascular disease may simply lower the threshold for clinical manifestation of AD pathology [64, 134, 143, 149]. Conversely, a history of hypertension or diabetes in patients with AD may bias clinicians towards a misdiagnosis of vascular dementia, underestimating the contribution of AD. The timing of several of the putative shared risk factors is of interest—coincident with the initial, presymptomatic deposition of Aβ in midlife (and in some studies associated with the extent of that presymptomatic deposition), rather than the maximum severity of disease as would be expected if the effects of these risk factors on CVD and AD were simply additive. Meta-analysis of data from three large-scale genome-wide association studies highlighted the involvement of cardiovascular disease-related pathways in AD [94].

Abnormalities broadly described as ‘cerebrovascular’ are common in AD. The relevant neuropathological literature was reviewed in detail by Attems and Jellinger [8]. In most cases of neuropathologically confirmed AD, post-mortem examination also reveals parenchymal vascular disease—both Aβ amyloid angiopathy and arteriolosclerotic small vessel disease (SVD) [75]. Cerebral infarcts or foci of haemorrhage can be demonstrated in over 50 % of AD brains, a figure that rises with age [46, 74, 75, 152]. Yet, other studies did not find any relationship between the burden of ischaemic vascular pathology and AD [31, 143].

Some studies suggest that AD is also associated with extracerebral atherosclerotic disease [65]. AD was associated with coronary artery disease in one study [151] but two other studies found no relationship between the severity of coronary artery disease and the Aβ plaque load [37, 86].

In several large post-mortem cohorts, AD was associated with atherosclerosis of the Circle of Willis [13, 67, 135, 172]. The age- and sex-adjusted severity of the atherosclerosis correlated with neuritic plaque [13, 67, 135, 172], tangle [13, 135, 172] and CAA severity scores [172], independent of *APOE* genotype [13, 172]. In contrast, there was no relationship between severity of the atherosclerosis and AD pathology in a series of elderly Japanese [73] or in the Baltimore Longitudinal Study of Aging cohort [37]. The explanation for the discrepant finding between these studies is not clear.

Two recent biomarker studies provided additional evidence of an association between CVD and AD. Hughes et al. [69] looked at the relationship between pulse wave velocity (an indicator of artery wall stiffness) and cerebral accumulation of Aβ over 2 years, as measured by positron emission tomography with Pittsburgh compound B, in 82 non-demented people aged 83 years or older. The authors found a significant relationship between vascular stiffness in larger central arteries (as indicated by carotid-femoral and heart-femoral pulse wave velocity) and cerebral Aβ accumulation over the two-year period. In another study, Kester et al. [83] analysed brain magnetic resonance imaging (MRI) findings and cerebrospinal fluid (CSF) tau and Aβ levels in 914 consecutive participants in the Amsterdam Dementia Cohort. The cohort included 30 people with a clinical diagnosis of vascular dementia, and 337 who had subjective memory complaints but were normal on cognitive testing. In both groups, white matter hyperintensities were associated with reduced Aβ42 in the CSF, as in AD. Microbleeds were associated with lower CSF Aβ42 in the vascular dementia group and higher CSF tau in the participants with subjective memory complaints. The associations were largely attributable to the *APOE* ϵ4 carriers in these groups.

## Structural disease of cerebral blood vessels in ageing in AD

Most patients with AD have Aβ amyloid angiopathy. Arteriolar Aβ amyloid angiopathy is demonstrable post-mortem in over 90 % of patients with AD compared with about 30 % of elderly controls [27, 44, 50, 95, 98, 101, 161, 162, 171]. The angiopathy predominantly affects the cerebral leptomeninges and cortex but sometimes also the cerebellum and, very occasionally, the brain stem. It tends to be more severe in AD than controls, particularly in *APOE* ϵ4-positive patients [27, 52, 131, 142]. In controls, arteriolar Aβ amyloid angiopathy is more strongly associated with *APOE* ϵ2 [95].

Accumulation of Aβ amyloid in cerebral arterioles may be accompanied by its accumulation in capillaries, a finding strongly associated with *APOE* ϵ4 and usually also with AD [1, 7, 95, 157]. In a series of 51 AD cases and 14 controls assessed independently by neuropathologists in three different centres, capillary amyloid angiopathy was detected in 35–45 % of the AD cases, predominantly in the occipital lobe, but in only a single control brain [95].

It is unclear whether or not patients with AD are more likely to have (non-amyloid) SVD. Numerous imaging and pathological studies have reported an increased prevalence of ‘small vessel disease’ in AD but most have conflated parenchymal ischaemic lesions—particularly in the white matter—with structural disease of cerebral blood vessels. *APOE* genotype influences the likelihood of ischaemic changes in AD. Morgen et al. [109] found that AD carriers of the *APOE* ϵ4 allele had a significantly greater volume of white matter hyperintensities than did non-carriers.

Whilst it is possible that many of the ischaemic lesions in patients with AD are attributable to cerebral SVD, some may have resulted from amyloid angiopathy or basal artery atherosclerosis, and a range of extracranial processes may also have contributed, including atherothrombotic or cholesterol emboli from carotid or aortic plaques, orthostatic hypotension [9, 81, 82, 150], cardiac valvular disease and cardiac arrhythmias [153], particularly atrial fibrillation [38, 123]. To try to address this, some of the studies excluded or adjusted for cardiovascular risk factors [140] but there remains a paucity of direct evidence linking SVD and AD, or indeed linking SVD and white matter lesions in AD: a post-mortem study of 125 cases of AD did not find any association between several objective measures of white matter damage (extent of immunolabelling for glial fibrillary acidic protein, axonal accumulation of Aβ precursor protein, axon density in superficial and deep white matter, intensity of staining for myelin) and severity of basal artery atherosclerosis, cerebral arteriolosclerosis or Aβ amyloid angiopathy, or *APOE* genotype [26].

Indirect evidence that SVD is more marked in AD comes from functional MRI analysis of regional cerebrovascular resistance (CVRi). In a study of CVRi in 12 patients with AD, 23 with MCI and 46 normal elderly controls, Nation et al. [114] found that cerebrovascular resistance was increased in several regions in AD and, to a lesser extent, in MCI. The increase was most marked in the thalamus and caudate nucleus, regions that are spared in Aβ amyloid angiopathy but have a predilection for SVD. Limitations of the study included the small cohort size and the use of non-contemporaneous measurements of peripheral rather than central blood pressure to calculate the CVRi, and in the absence of neuropathological confirmation the precise substrate of the increase in vascular resistance remains a matter of speculation.

Several studies have reported that the degeneration of cerebral capillaries in AD exceeds that in age-matched controls [10, 25, 70, 144]. The degeneration affects both endothelial cells and pericytes and leads eventually to an increase in the number of ‘string’ vessels consisting solely of tubes of collagen. This is not accompanied by a compensatory increase in the formation of new capillaries [11, 70, 158]. The degeneration of pericytes and endothelial cells is probably interrelated.

## CVD exacerbates neurological dysfunction and brain damage in AD

**Fig. 1:**
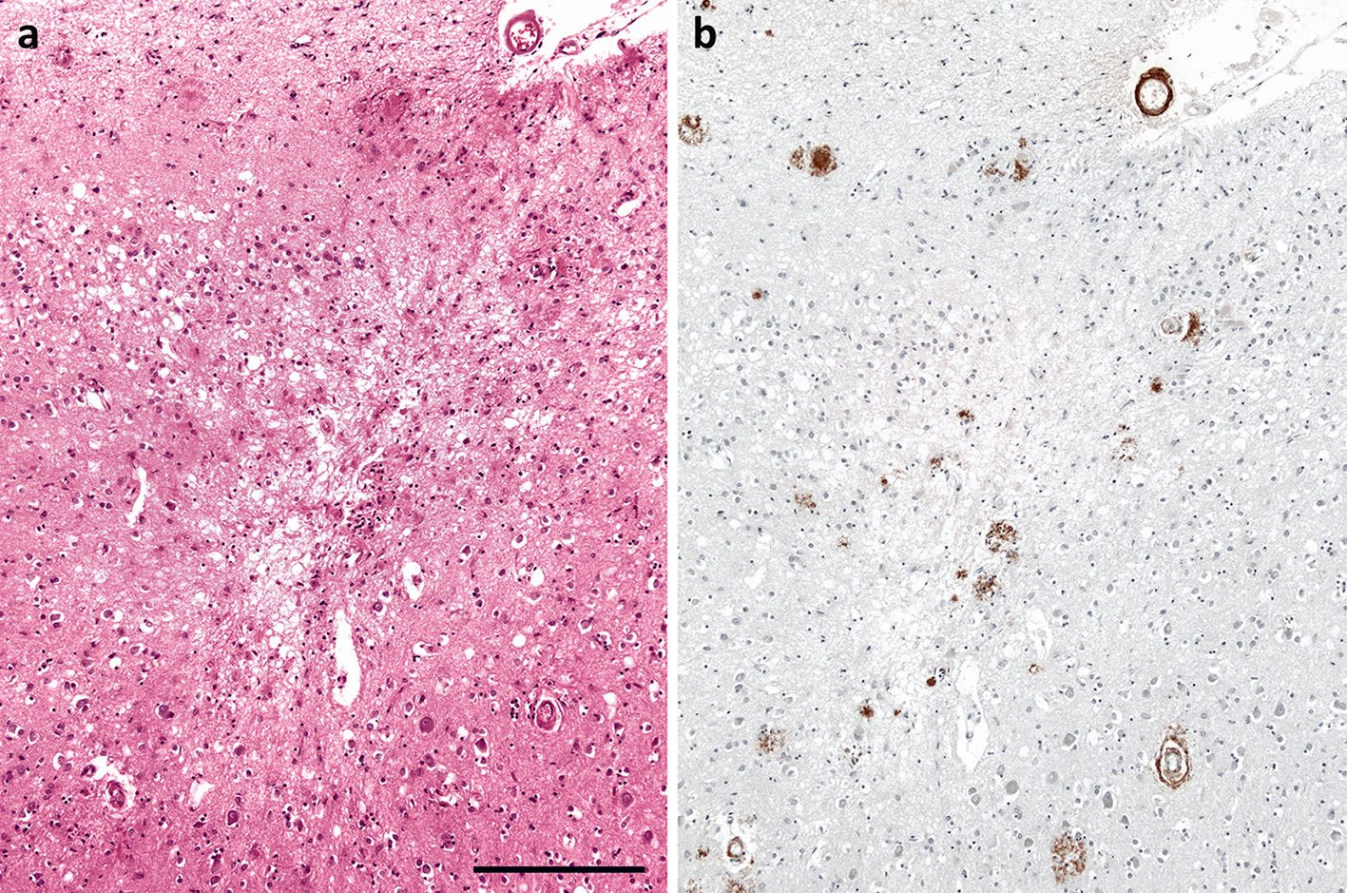
Infarction associated with Aβ amyloid angiopathy. **a** Microinfarct in cerebral cortex. The lesion is rarefied and gliotic, and includes a few macrophages. **b** Aβ immunohistochemistry on an adjacent section revealed moderately severe Aβ amyloid angiopathy. Note the circumferential deposition of Aβ in a sulcal arteriole overlying the microinfarct. *Bar* 250 μm

The most obvious impact of CVD is on cerebral perfusion. There is a substantial literature documenting the association of Aβ amyloid angiopathy and SVD with infarcts (Fig. 1) and other ischaemic abnormalities [21, 23, 32, 33, 43, 45, 121, 122, 133, 134, 136, 161]. The mechanisms include susceptibility to thrombosis [21, 97], permanent reduction in blood flow as a result of reduced calibre of more severely affected blood vessels, and impaired modulation of the calibre in response to haemodynamic stress and alteration in metabolic demand.

Patients with AD have impaired autoregulation (i.e. reduced ability to maintain cerebral perfusion when perfusion pressure changes, usually as a result of hypotension) and reduced vasomotor reactivity to hypercapnia [36]. Patients with probable Aβ amyloid angiopathy (diagnosed according to the Boston criteria [84]) also show neurovascular decoupling, as evidenced by impaired functional hyperaemia [39, 130] (i.e. a reduced regional increase in blood flow in response to increased local metabolic demand). Similar abnormalities in regulation of cerebral blood flow were demonstrated in mouse models of cerebral Aβ accumulation [71, 117] and Aβ amyloid angiopathy [129, 147]. In patients with Aβ amyloid angiopathy, reduced primary visual cortex fMRI responsiveness to a visual task correlated with the volume of white matter hyperintensities, an indicator of the severity of white matter ischaemia [130].

**Fig. 2:**
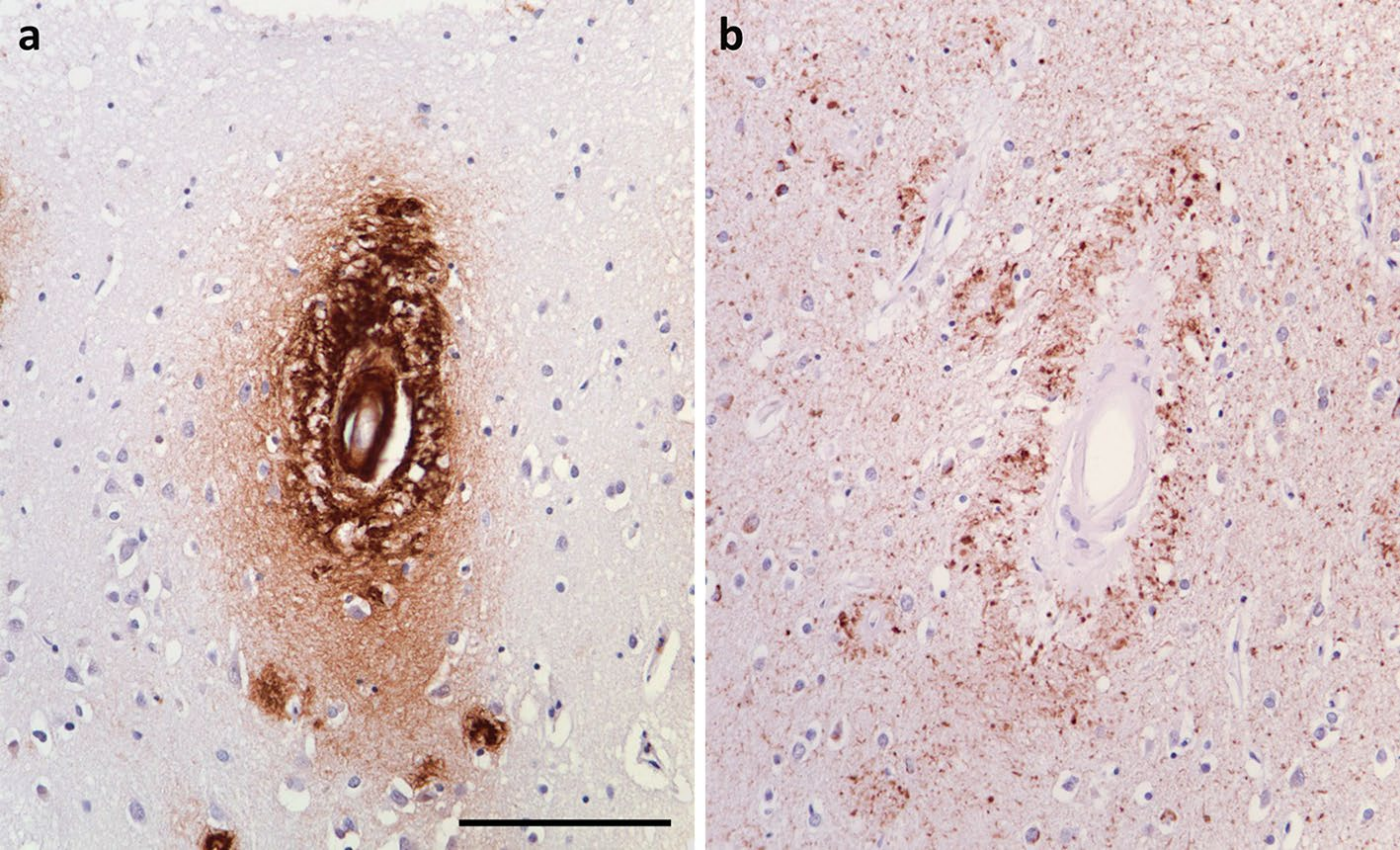
Perivascular tau associated with Aβ amyloid angiopathy. **a** Arteriolar and dyshoric deposition of Aβ in a patient with severe amyloid angiopathy. **b** Immunolabelling of an adjacent section for phospho-tau showed accentuated accumulation of tau around the affected arteriole. *Bar* 250 μm

In a series of studies of human brain tissue and animal models, Weller, Carare, Hawkes and colleagues showed that interstitial fluid drains from the brain along perivascular spaces and basement membranes [164, 174], and that Aβ within the interstitial fluid tends to precipitate within the perivascular basement membranes [165, 166]. Further evidence that Aβ amyloid angiopathy reflected the precipitation of Aβ from the interstitial fluid came from the finding of Jucker and colleagues that neuron-specific overexpression of Aβ precursor protein caused the development of Aβ amyloid angiopathy in APP23 mice [22, 63]. Vascular deposition of Aβ is increased by possession of *APOE* ϵ4 [27]; this is probably partly related to elevation of the ratio Aβ40:42 [48, 63] and partly to alterations in the composition of the perivascular basement membranes [60]. Vascular deposition of Aβ is also increased if drainage is impeded by Aβ amyloid angiopathy or by age-related changes to the perivascular basement membranes [58, 59, 76, 166]. The full range of downstream effects of impeded drainage of solutes in Aβ amyloid angiopathy remains to be determined. One probable consequence is an increase in the phosphorylation of tau, as evidenced by the more abundant neurofibrillary pathology around Aβ-laden than non-Aβ-laden parenchymal arterioles in AD (Fig. 2) [169]. A similar increase in neurofibrillary pathology was demonstrated adjacent to parenchymal arterioles in the amyloid angiopathy of familial British dementia, in which there is vascular deposition of ABri [66], which, like vascular Aβ, probably impedes the drainage of interstitial fluid.

Other ways in which AD-associated structural vascular disease exacerbates neurological dysfunction and brain damage include increased susceptibility to haemorrhage and, possibly, impaired blood–brain barrier (BBB) function. Carrano et al. [24] noted the loss of several components of endothelial tight junctions in Aβ-laden capillaries in post-mortem brain tissue from patients with Aβ amyloid angiopathy, and similar findings were documented in several in vitro and ex vivo experimental studies, in which exposure of endothelial cells to Aβ40 or Aβ42 caused loss or mislocation of tight junction proteins [57, 85, 100, 154]. Hartz et al. [57] reported that 2 of 19 patients who fulfilled clinical criteria for probable Aβ amyloid angiopathy had MRI evidence of BBB leakage and posterior reversible encephalopathy syndrome.

Degeneration of pericytes probably contributes to the impairment of BBB function in AD. In mice pericytes were shown to be needed for maintenance of the blood–brain barrier and capillary perfusion [3, 14]. Sengillo et al. [144] found the loss of pericytes in cerebral cortex and hippocampus from AD patients to correlate with the extent of disruption of the blood–brain barrier, as demonstrated by extravasation of immunoglobulin G and perivascular deposition of fibrin. In contrast, a study of a range of mouse models of AD did not find increased permeability of the BB to antibodies [17].

**Fig. 3:**
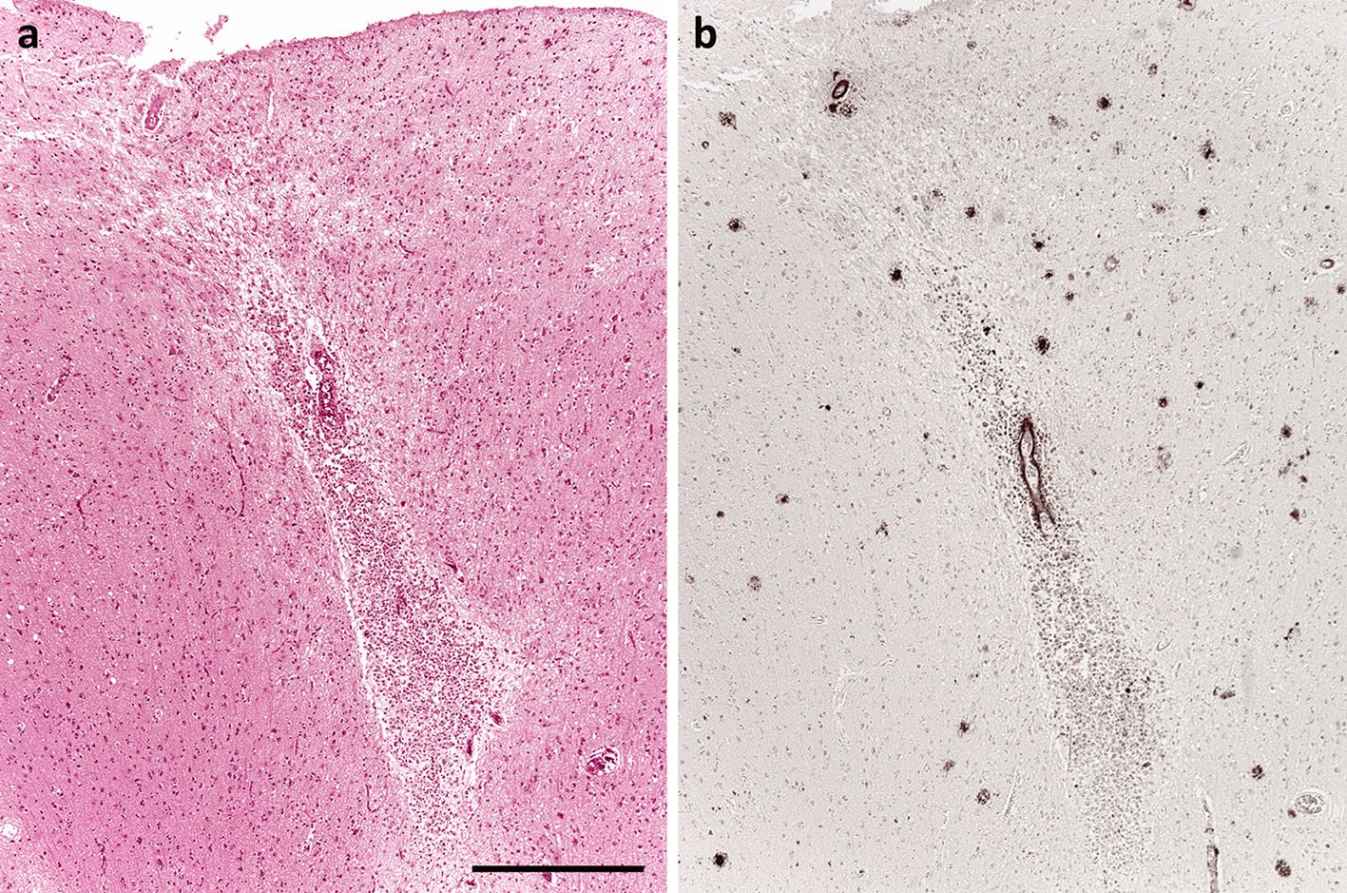
Microhaemorrhage associated with Aβ amyloid angiopathy. **a** Accumulation of numerous haemosiderin-laden macrophages around a cortical arteriole with a strongly eosinophilic wall. **b** The arteriole is immunopositive for Aβ, as demonstrated in this adjacent section. *Bar* 500 μm

Numerous studies have documented that patients with Aβ amyloid angiopathy are prone to cerebral haemorrhage, particularly lobar haemorrhage and cortical microhaemorrhage (Fig. 3) [43, 50, 51, 97, 111, 115, 131, 161, 171]. Both the ε2 and ε4 alleles of *APOE* increase the risk of Aβ amyloid angiopathy-associated lobar haemorrhage [52, 53, 116]; ε4 may do so by promoting the accumulation of Aβ in the walls of arterioles, and ε2 by promoting the development of vasculopathic changes, particularly fibrinoid necrosis, in the amyloid-laden vessels [54, 103]. Patients with Aβ amyloid angiopathy are also prone to develop superficial cortical siderosis [29, 93].

## Cerebral hypoperfusion is an early feature of AD

**Fig. 4:**
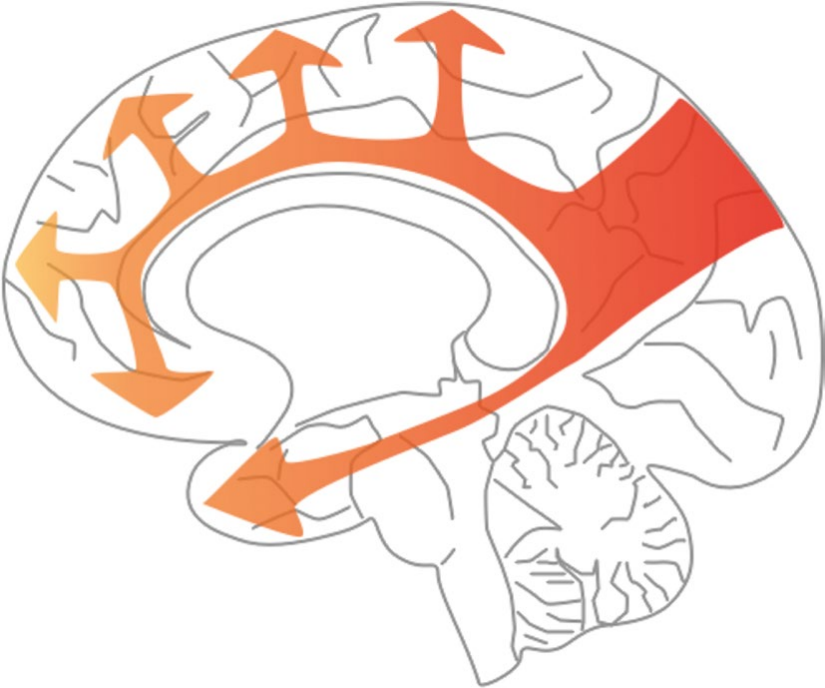
Stereotyped pattern of spread of hypoperfusion in AD. The *arrows in this diagram* indicate the progression of hypoperfusion, starting from the precuneus, well before the onset of dementia, and spreading to the rest of the parietal cortex and the cingulate gyrus, then the frontal and temporal cortex, largely sparing the occipital cortex until late disease

Cerebral hypoperfusion is demonstrable several years before the onset of clinical AD. Imaging studies in people with MCI [6, 18, 34], healthy carriers of the *APOE* ϵ4 allele [89] and those with gene mutations known to cause autosomal dominant AD [15] have shown that the distribution and spread of the hypoperfusion are highly stereotyped, and mirror the accumulation of Aβ amyloid within the brain parenchyma, particularly during the preclinical phases of disease. The first affected region is the precuneus, about 10 years before the development of dementia (Fig. 4). From there the hypoperfusion spreads to involve the cingulate gyrus and the lateral part of the parietal lobe, then the frontal and temporal lobes and eventually most of the cerebrum.

At least in preclinical early AD, the primary abnormality is one of inadequate blood supply, not reduced metabolic demand, in that the reduction in oxygenation is not commensurate with the decline in blood flow. Several fMRI studies have found that the regional oxygen extraction fraction (rOEF) is significantly elevated in AD, particularly in the parietal cortex [112, 113, 159], and Tarumi et al. [156] found the tissue oxygenation index (TOI, a measure of the saturation of haemoglobin by oxygen, determined by near infrared spectroscopy) to be significantly increased in the cortex in people with amnestic MCI. Were the decline in blood flow to be a response to decreased metabolic demand, the rOEF would be reduced and the TOI increased.

**Fig. 5:**
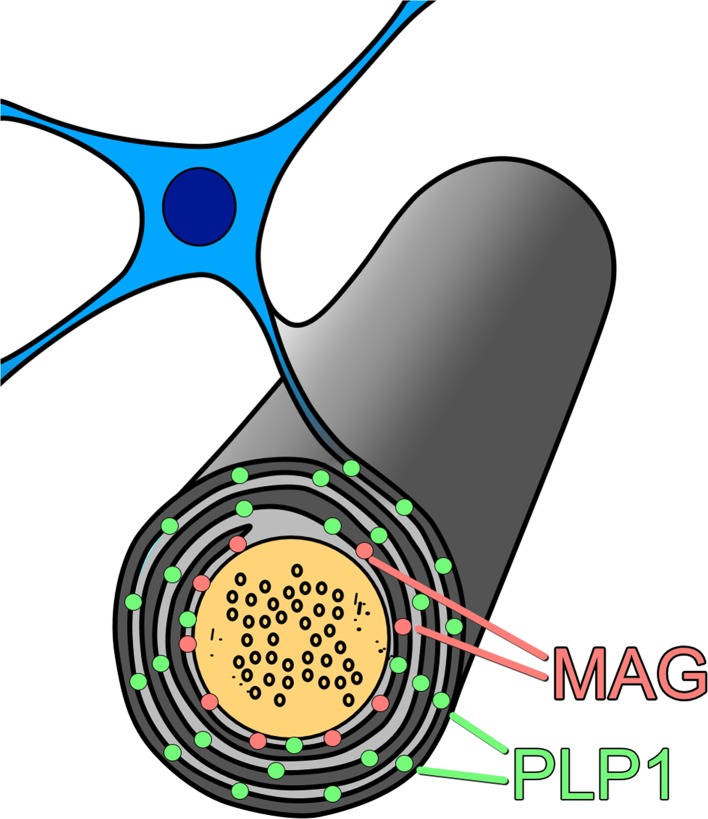
Schematic illustration of the distribution of MAG (*Pink dots*) and PLP1 (*Green dots*) in the myelin sheath. When the supply of oxygen and glucose is insufficient to meet the metabolic needs of the oligodendrocyte, as occurs in hypoperfusion, the first part of the cell to degenerate is the adaxonal loop of myelin—the part of the oligodendrocyte that is furthest away from the cell body (so-called dying-back oligodendrogliopathy). Because MAG is restricted to the adaxonal loop of myelin whereas PLP1 is widely distributed throughout the myelin sheath, hypoperfusion leads to greater loss of MAG than PLP1. In contrast, degeneration of nerve fibres causes loss of both MAG and PLP1. The severity of ante-mortem hypoperfusion can be assessed by measuring the MAG:PLP1 ratio

We recently developed a post-mortem biochemical method to study hypoperfusion in brain tissue [11, 12, 96]. The method is based on the differential susceptibility of different myelin proteins to ischaemia. The myelin proteins are synthesised in the oligodendrocyte cell body and require energy-dependent transport to reach their sites of insertion into the myelin sheath. Myelin-associated glycoprotein (MAG) is one of the myelin proteins inserted furthest away from the cell body, in the adaxonal loop of myelin, the first part of the sheath to degenerate when blood supply is insufficient to meet the energy demands of the oligodendrocyte. Measurement of the ratio of MAG to another myelin protein such as proteolipid protein-1 (PLP1), present throughout the myelin sheath, indicates the extent to which the blood supply meets the energy requirements of the oligodendrocyte (Fig. 5).

In the deep parietal white matter in an elderly cohort that included AD, VaD and cognitively normal cases, MAG:PLP1 showed a highly significant negative correlation with severity of SVD and a highly significant positive correlations with the concentration of the vasoconstrictor endothelin-1 (EDN1) and with the concentration of vascular endothelial growth factor (VEGF) [11, 12]. These biochemical relationships were confirmed in deep frontal white matter in an Oxford cohort that had excluded brains with more than mild AD or Lewy body pathology [12]. Our interpretation was that as SVD severity increased, EDN1 production had been downregulated and VEGF production upregulated in response to the reduced tissue oxygenation.

Having established the utility of this post-mortem biochemical assessment of ante-mortem oxygenation of brain tissue, we examined mid-frontal cortex from AD and control brains, to determine whether there was evidence that any reduction in perfusion exceeded the decline in metabolic demand, and to try to identify possible mediators of hypoperfusion [158]. Our findings confirmed that perfusion was indeed pathologically reduced in AD. More recently, we examined precuneus, the region first affected by hypoperfusion in AD [106]. MAG:PLP1 in this region was reduced ~50 % in early AD (Braak stage III–IV). Although MAG:PLP1 in the precuneus remained low in advanced AD (stage V–VI), the reduction was less pronounced, possibly reflecting falling oxygen demand.

## The main cause of cerebral hypoperfusion in AD is probably non-structural vascular dysfunction rather than structural pathology

Although structural disease of blood vessels contributes to cerebral hypoperfusion in at least some patients with AD, we did not find Aβ amyloid angiopathy, SVD or reduced microvessel density to contribute significantly to the reduction in MAG:PLP1 in the frontal cortex, medial parietal cortex or parietal white matter in most cases [11, 106, 158]. It is of note that the consistent distribution of hypoperfusion in early AD, involving the precuneus and, to a lesser extent, the cingulate gyrus, is not in a watershed region, as might be expected if postural hypotension or basal atheroma were responsible. If SVD were the explanation, the hypoperfusion would be more likely to involve the deep white matter and basal ganglia. Lastly, the distribution of Aβ amyloid angiopathy is quite variable but it tends to be most severe in the occipital region, which is affected by hypoperfusion only in the late stages of AD.

There is a range of potential non-structural substrates of hypoperfusion in AD, several of which involve pathways that are strongly influenced by Aβ. Elevated Aβ was previously shown to cause vasoconstriction, reduce cerebral perfusion and prevent functional hyperaemia and autoregulation in mice transgenic for mutant human APP [71, 117, 118, 129]; to increase expression of endothelin-converting enzyme-1 and -2 (ECE1 and ECE2) by human cerebrovascular endothelial cells and neuroblastoma cells, respectively, leading to elevated EDN1 production [126–128]; to inhibit endothelial nitric oxide synthase activity [49, 88], reducing endothelial production of the vasodilator nitric oxide; and to increase the neuronal expression and activity of angiotensin-converting enzyme (ACE) [107], which catalyses the production of the vasoconstrictor angiotensin II (AngII). Support for the pathogenic relevance of several of these experimental observations comes from the demonstration of elevated EDN1 level and ACE activity in post-mortem temporal and frontal cortex from patients with AD [105, 127, 158]. Aβ is a substrate of ECE1 and ECE2 [40–42, 124] and also of ACE [62, 68, 120], and it seems possible that the upregulation of production of EDN1 and AngII may be a side effect of over-activation of these enzyme systems by the accumulation of substrate.

**Fig. 6:**
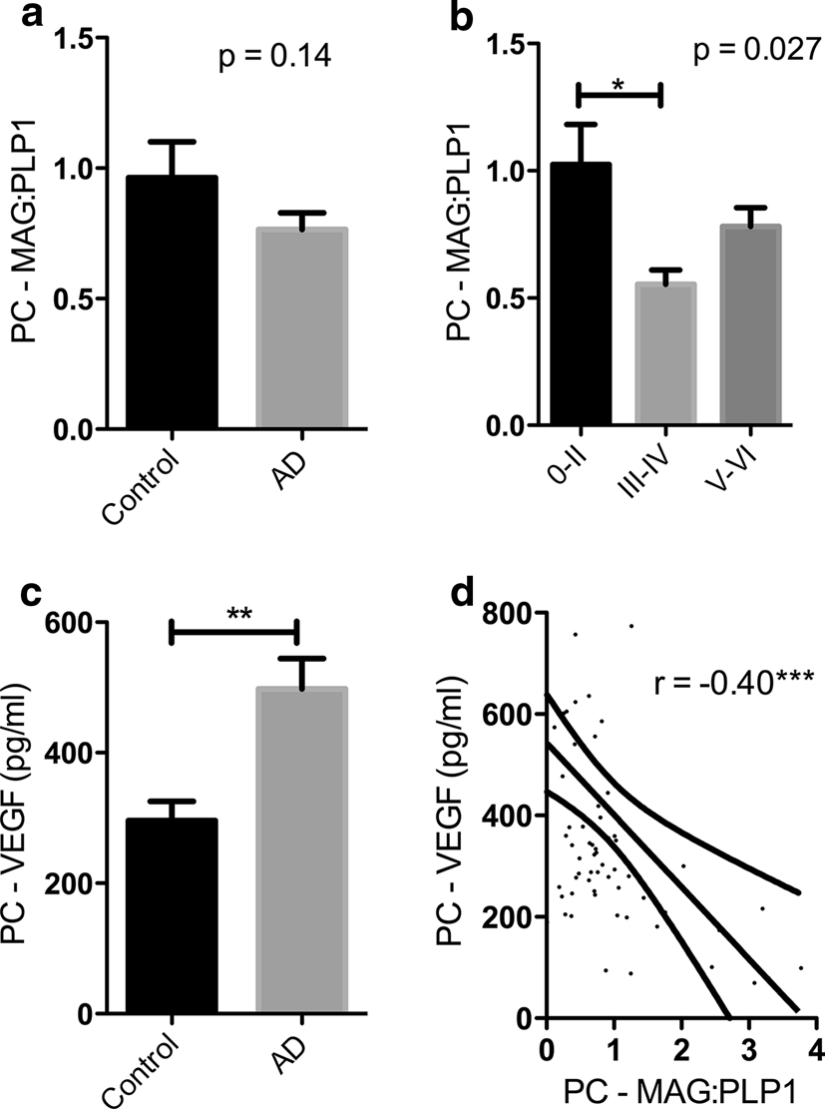
Oxygenation of the precuneus is reduced in early Alzheimer’s disease. **a** *Bar chart* showing a reduction in the ratio of myelin glycoprotein (MAG) to proteolipid-1 protein (PLP-1) (MAG:PLP1) in the precuneus in AD. The *bars* indicate the mean and SEM. **b** Bar chart showing marked variation in MAG:PLP1 (*P* = 0.027) with disease stage. For this analysis, control and AD cases were combined and grouped according to Braak tangle stage (0–II, III–IV and V–VI). Post hoc analysis revealed that MAG:PLP1 was significantly reduced in early AD (Braak stage III–IV) compared to controls (*P* = 0.027). **c** Bar chart showing elevated vascular endothelial growth factor (VEGF), an independent marker of cerebral perfusion, in AD. **d** Scatterplot showing the highly significant negative correlation between MAG:PLP1 and VEGF concentration in the precuneus (*r* = −0.40, *P* = 0.0007). The best-fit linear regression line and 95 % confidence interval are superimposed. **P < 0.001, ***P < 0.0001. Reproduced with permission from [106]

**Fig. 7:**
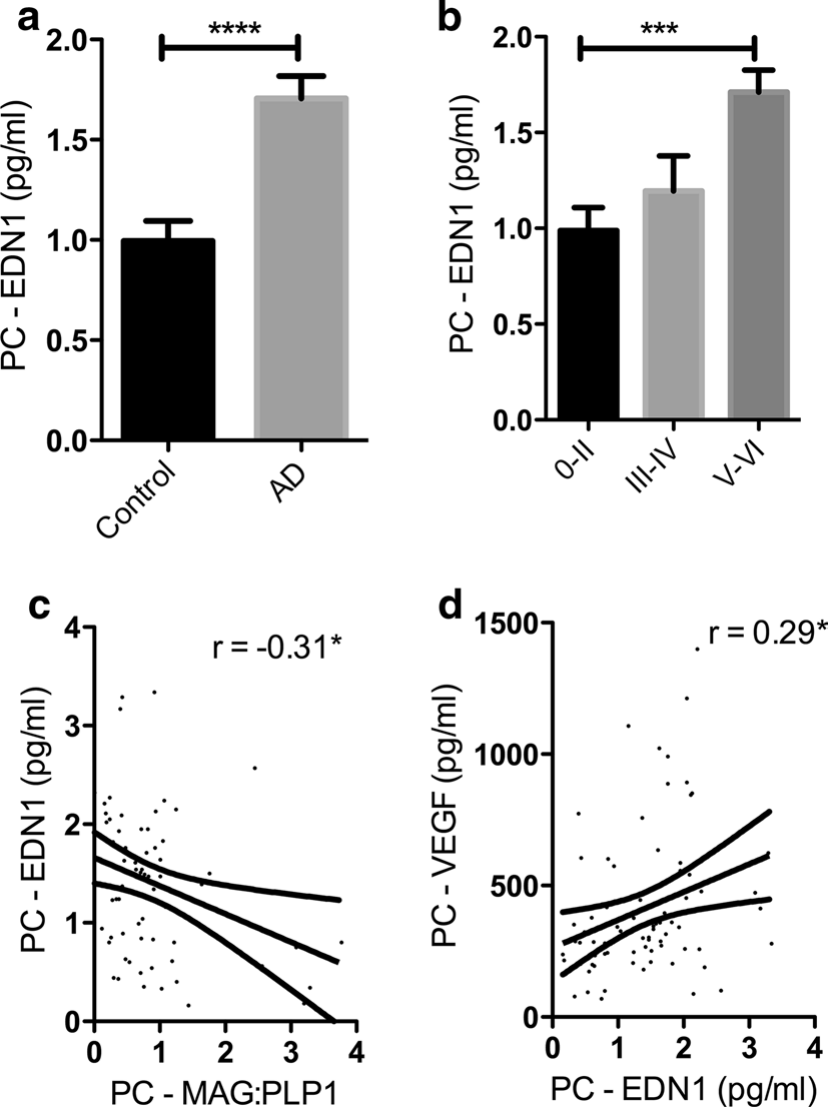
Reduced oxygenation of the precuneus in AD is associated with elevated endothelin-1 (EDN1). **a** *Bar chart* showing significantly increased EDN1 in AD within the precuneus. **b** *Bar chart* showing increased EDN1 levels in relation to disease severity when control and AD cases were subdivided according to Braak tangle stage (0–II, III–IV and V–VI) irrespective of the presence or absence of a history of dementia. Scatterplots showing the inverse correlation between EDN1 concentration and MAG:PLP1 ratio (*r* = −0.31) (**c**), and the positive correlation between EDN1 and VEGF (*r* = 0.29) (**d**). *P < 0.05, ***P < 0.001, ****P < 0.0001. Reproduced with permission from [106]

More recently, we looked at MAG:PLP1, SVD, Aβ amyloid angiopathy, EDN1 level, ACE level and activity, and AngII level in precuneus from post-mortem human brains showing a spectrum of severity of AD (as indicated by Braak tangle stage). We showed that MAG:PLP1 declined in the precuneus even in early AD (i.e. in Braak stage III–IV disease) (Fig. 6). Indeed, despite progressive elevation in EDN1 concentration with disease progression, the MAG:PLP1 ratio was lower in early than in late AD (Fig. 7), presumably reflecting falling metabolic demand with increasing synaptic and neuronal damage. In the precuneus, unlike in the frontal cortex in advanced AD, we did not find elevation of ACE or AngII.

EDN1 in the cortex correlated highly significantly with the levels of both soluble and insoluble Aβ42, which we showed previously to upregulate neuronal ECE2 [126], but not with Aβ40, which we found to upregulate endothelial ECE1 [127]. The findings suggest that reduced oxygenation of the precuneus in early AD is likely to result, at least in part, from Aβ42-mediated upregulation of ECE2.

MAG:PLP1 was also reduced in the parietal white matter in AD but here the decline correlated positively with the level of EDN1, in keeping with a protective vasodilatory response to reduced oxygenation. However, the decline of MAG:PLP1 in the white matter was associated with increasing EDN1 in the overlying cortex, perhaps reflecting EDN1-mediated vasoconstriction of perforating arterioles, which traverse the cortex to perfuse the white matter.

Although the main focus of studies of functional rather than structural vascular abnormalities has been on changes in arteriolar calibre, biochemical alterations in AD may also affect vascular permeability. In particular, several lines of evidence indicate that Aβ upregulates the plasma kallikrein-kinin system, leading to the production of bradykinin, which causes dilatation and increased permeability of venules and, at higher concentration, dilatation of arterioles as well [2]. The activating enzyme, plasma kallikrein, circulates as an inactive zymogen and is activated through a cleavage process involving Factor XIIa. Active plasma kallikrein cleaves its major substrate, high molecular weight kininogen (HK) to liberate bradykinin and activated HK. Aβ was shown to stimulate the production of bradykinin by endothelial cells [16, 146, 170]. Cerebroventricular infusion of Aβ40 in rats caused a 10-fold increase of bradykinin in the CSF and accumulation of degradation fragments of bradykinin in brain tissue [72]. Ashby et al. [5] demonstrated that plasma kallikrein mRNA was significantly increased in the frontal cortex in AD, and plasma kallikrein activity was significantly increased in the frontal and temporal cortex in AD.

## Cerebral hypoperfusion probably accelerates the progression of AD

As noted above, ischaemic brain damage is common in AD and in many cases contributes to the cognitive impairment. Ischaemia may also contribute to the progression of AD itself. Ischaemia in animal models, or its in vitro stimulation, is associated with increased production of Aβ42 (for reviews see [31, 96]). Acute cerebral ischaemia or hypoxia caused overexpression of APP mRNA [145], upregulation of BACE1 mRNA and protein, increased β-secretase activity and the production of Aβ peptide [55, 91, 167, 175]. BACE1 and Aβ (but not APP) level were also elevated in a chronic hypoperfusion model [176]. Transient ischaemia increased PSEN1 mRNA in the gerbil hippocampus [155] and repeated episodes of hypoxia enhanced β-secretase-mediated cleavage of APP, and the level of APH-1a, another component of γ-secretase complex [91]. In other in vitro studies, hypoxia and/or oxidative modification reduced the activity of the Aβ-degrading enzymes neprilysin and insulin-degrading enzyme [47, 148, 163]. Lastly, transient cerebral ischaemia caused hyperphosphorylation of tau in cortical neurons [168].

Although most evidence that cerebral hypoperfusion may accelerate the progression of AD comes from in vitro and animal studies, some clinical observations support this possibility. In several clinical studies of cerebral blood flow and glucose utilisation in patients with a clinical diagnosis of AD or MCI, hypoperfusion predicted the development or rate of cognitive decline [19, 20, 28]. Patients who survived transient cerebral hypoperfusion as a result of cardiac arrest had elevated serum Aβ42 over several days [173]. And whilst that might be explained by non-specific ‘leakage’ of Aβ42 from damaged brain tissue, the same explanation could not be applied to the sustained elevation of serum Aβ42 in a study of patients with diffuse TBI with brain swelling and presumably at least some hypoperfusion, as they showed a concomitant reduction of Aβ42 in the CSF [108].

## Implications for therapy

The identification of functionally important non-structural abnormalities of the cerebral vasculature in ageing and dementia, particularly in AD, raises the prospect that pharmacotherapy targeting the relevant biochemical pathways might slow the progression of dementia, by improving cerebral perfusion and reducing permeability of the BBB. An obvious potential target is the endothelin system, for which there are already several well-tolerated antagonists. Bosentan, a non-selective endothelin receptor (EDNR) antagonist [138], has been licenced since 2001 for the treatment of pulmonary hypertension, another disease in which there is upregulation of ECEs and increased production of EDN1 [132, 137]. Bosentan produces significant, clinically useful, sustained improvement in pulmonary blood flow and exercise tolerance in patients with pulmonary hypertension. Bosentan was also shown to prevent the attenuation of endothelium-dependent aortic and carotid vasodilatation that is seen in Tg2576 mice (which overexpress a mutant form of APP bearing the Swedish double mutation). However, EDN1 is thought to act on EDNRA receptors (the predominant EDN1 receptor type in cerebral vessels) to reduce CBF in AD, and there are selective EDNRA receptor antagonists such as zibotentan [56, 110] which offer potential advantages over non-selective EDNR antagonists [125]. Other targets might include the plasma kallikrein-kinin [5] and renin-angiotensin systems [78]. The therapeutic potential of inhibiting the renin-angiotensin system in AD is attracting a great deal of interest and clinical investigation [4, 77, 79, 80, 119].
